# Clinical Presentation, Management, and Evolution of Lymphomas in Patients with Inflammatory Bowel Disease: An ENEIDA Registry Study

**DOI:** 10.3390/cancers15030750

**Published:** 2023-01-25

**Authors:** Ivan Guerra, Luis Bujanda, Miriam Mañosa, Isabel Pérez-Martínez, María José Casanova, Luisa de la Peña, Marina de Benito, Montserrat Rivero, Pilar Varela, Lorena Bernal, Ana Carolina Franco, Yolanda Ber, Marta Piqueras, Carlos Tardillo, Ángel Ponferrada, Sonsoles Olivares, Alfredo J. Lucendo, Pau Gilabert, Mónica Sierra Ausín, María Bellart, Amaia Herrarte, Margalida Calafat, Ruth de Francisco, Javier P. Gisbert, Jordi Guardiola, Eugeni Domènech, Fernando Bermejo

**Affiliations:** 1 Gastroenterology Unit, Hospital Universitario de Fuenlabrada, 28942 Madrid, Spain; 2 Gastroenterology Unit, Hospital Donostia/Instituto Biodonostia, Universidad del País Vasco UPV/EHU, 20014 Donostia-San Sebastián, Spain; 3 Centro de Investigación Biomédica en Red de Enfermedades Hepáticas y Digestivas (CIBEREHD), 28029 Madrid, Spain; 4 Gastroenterology Unit, Hospital Universitari Germans Trias i Pujol, 08916 Badalona, Spain; 5 Gastroenterology Unit, Hospital Universitario Central de Asturias, and Instituto de Investigación Sanitaria del Principado de Asturias (ISPA), 33011 Oviedo, Spain; 6 Gastroenterology Unit, Hospital Universitario de La Princesa, IIS-Princesa, Universidad Autónoma de Madrid UAM, 28006 Madrid, Spain; 7 Gastroenterology Unit, Hospital Universitari de Bellvitge, Institut d’Investigació Biomèdica de Bellvitge (IDIBELL), Universitat de Barcelona, l’Hospitalet de Llobregat, 08907 Barcelona, Spain; 8 Gastroenterology Unit, Hospital Universitario Río Hortega, 47012 Valladolid, Spain; 9 Gastroenterology Unit, Hospital Universitario Marqués de Valdecilla and IDIVAL, 39008 Santander, Spain; 10 Gastroenterology Unit, Hospital Universitario de Cabueñes, 33394 Gijón, Spain; 11 Gastroenterology Unit, Hospital General Universitario Dr Balmis de Alicante, 03010 Alicante, Spain; 12 Onco-Hematology Unit, Hospital Universitario de Fuenlabrada, 28942 Madrid, Spain; 13 Gastroenterology Unit, Hospital General San Jorge, 22004 Huesca, Spain; 14 Gastroenterology Unit, Consorci Sanitari Terrassa, 082217 Terrassa, Spain; 15 Gastroenterology Unit, Hospital Universitario Nuestra Señora de la Candelaria, 38010 Tenerife, Spain; 16 Gastroenterology Unit, Hospital Universitario Infanta Leonor, 28031 Madrid, Spain; 17 Gastroenterology Unit, Hospital 12 de Octubre, 28041 Madrid, Spain; 18 Gastroenterology Unit, Hospital General de Tomelloso, Ciudad Real, Spain and Instituto de Investigación Sanitaria de Castilla-La Mancha (IDISCAM), 13700 Tomelloso, Spain; 19 Gastroenterology Unit, Hospital de Viladecans, 08840 Barcelona, Spain; 20 Gastroenterology Unit, Complejo Asistencial Universitario de León, 24071 León, Spain

**Keywords:** inflammatory bowel disease, lymphoma, thiopurines, anti-TNF

## Abstract

**Simple Summary:**

An increased risk of hematological malignancies, mainly lymphomas, has been described in patients with inflammatory bowel disease (IBD). Because there are scarce data about the management and evolution of lymphomas in patients with IBD, the aim of our study was to analyze these points in those patients with IBD and lymphoma diagnosis included in the prospectively maintained ENEIDA registry of GETECCU. We identified 52 patients (2.4 cases of lymphoma/1000 patients with IBD). We found that most IBD patients had been treated with thiopurines and/or anti-TNF agents before lymphoma diagnosis, and these patients were younger at diagnosis of lymphoma than those not treated with these drugs. Relapse and mortality of lymphoma were not related with these therapies. The five-year survival rate was 85% for non-Hodgkin lymphoma and 84% in patients with Hodgkin lymphoma.

**Abstract:**

An increased risk of lymphoma has been described in patients with inflammatory bowel disease (IBD). The aims of our study were to determine the clinical presentation, the previous exposure to immunosuppressive and biologic therapies, and the evolution of lymphomas in patients with IBD. IBD patients with diagnosis of lymphoma from October 2006 to June 2021 were identified from the prospectively maintained ENEIDA registry of GETECCU. We identified 52 patients (2.4 cases of lymphoma/1000 patients with IBD; 95% CI 1.8–3.1). Thirty-five were men (67%), 52% had ulcerative colitis, 60% received thiopurines, and 38% an anti-TNF drug before lymphoma diagnosis. Age at lymphoma was lower in those patients treated with thiopurines (53 ± 17 years old) and anti-TNF drugs (47 ± 17) than in those patients not treated with these drugs (63 ± 12; *p* < 0.05). Five cases had relapse of lymphoma (1.7 cases/100 patient-years). Nine patients (17%) died after 19 months (IQR 0–48 months). Relapse and mortality were not related with the type of IBD or lymphoma, nor with thiopurines or biologic therapies. In conclusion, most IBD patients had been treated with thiopurines and/or anti-TNF agents before lymphoma diagnosis, and these patients were younger at diagnosis of lymphoma than those not treated with these drugs. Relapse and mortality of lymphoma were not related with these therapies.

## 1. Introduction

Inflammatory bowel disease (IBD), including Crohn’s disease (CD), ulcerative colitis (UC), and unclassified colitis, has been associated with an increased risk of intestinal and extraintestinal cancers [1,2]. A higher risk of developing hematological malignancies has been described in patients with IBD compared with the general population; leukemia in patients with UC, and lymphoma, mainly non-Hodgkin lymphoma (NHL), in those patients with CD [1]. Early IBD onset, male gender, and age >65 years old are risk factors for hematological malignancies in IBD patients [1]. In a recent meta-analysis, an increased risk of hematological cancer in general was found in CD patients, and especially of lymphomas in patients with CD, with a borderline significance for specific risk in NHL and leukemia [2]. The state of immune activation associated with IBD and some therapies used in IBD, such as thiopurines and anti-TNF drugs, have been described as possible factors that contribute to the increased risk of lymphomas in patients with IBD [1,3,4]. There are scarce data about the clinical presentation, management, and evolution of lymphomas in patients with IBD. The aims of the present study were to determine the clinical presentation of lymphoma, the previous exposition to immunosuppressive and biological therapies, and the management and evolution of lymphomas in patients with IBD.

## 2. Materials and Methods

### 2.1. Data Source

This was an observational study including patients from the Spanish ENEIDA registry. The ENEIDA registry (Nationwide study on genetic and environmental determinants of inflammatory bowel disease) is a nationwide Spanish project of IBD patients promoted by GETECCU (Spanish Working Group in Crohn’s Disease and Ulcerative Colitis) [5]. The ENEIDA registry started including patients in October 2006. The database is prospectively maintained under continuous external monitoring with respect to completeness and the consistency of the entered data, but only local investigators can introduce and modify the data.

### 2.2. Study Population

IBD patients included in the ENEIDA registry with diagnosis of lymphoma from October 2006 to June 2021 were included in the study. Lymphoma diagnosis was determined following current international criteria [6].

### 2.3. Variables of Interest

The registry prospectively records the gender, age, ethnic group, smoking status, age at diagnosis of IBD, type of IBD, location of disease, need for admission or surgery, treatments for IBD including immunomodulator and biological treatments with dates of start and withdrawal of each of these drugs and date of last evaluation. Additional information about lymphomas provided by the investigators of each participating center includes type of lymphoma, date of diagnosis, stage following the Lugano classification [7], clinical presentation, diagnostic test, treatment, relapse, and consensus about the management with hematology. These additional data were collected and managed using REDCap electronic data capture tools hosted at the Asociación Española de Gastroenterología (AEG; www.aegastro.es; accessed on 1 June 2022) [8,9]. We defined two NHL subtypes by rate of progression (aggressive and indolent) following the World Health Organization’s (WHO) classification [6]. Diffuse large B-cell lymphoma, mantle cell lymphoma, plasmablastic lymphoma, and cutaneous lymphoma were defined as aggressive NHL. Indolent NHL included follicular, marginal zone, and lymphoplasmacytic lymphoma.

### 2.4. Statistical Analysis

For quantitative variables, data are shown as mean and standard deviation or as median and interquartile range (IQR), according to the presence or absence of a normal distribution, respectively. Comparison between categorical variables was performed using the Chi-square test. Continuous variables were compared using Student’s *t*-test or the nonparametric Wilcoxon’s signed rank test as appropriate. Statistical significance was considered for *p* values lower than 0.05. Survival since lymphoma diagnosis was estimated using Kaplan–Meier curves for aggressive NHL, indolent NHL, and Hodgkin lymphoma, being the curves compared with the log-rank test. Cox regression analysis was performed to identify the potential risk factors for relapse of lymphoma and mortality. The independent variables included in the Cox regression analysis were age at diagnosis of IBD and lymphoma, gender, type of IBD (CD or UC), smoking status, specific type of lymphoma (NHL vs. Hodgkin lymphoma), and treatment with thiopurines and anti-TNF drug therapy.

## 3. Results

We identified 52 patients with lymphoma in 18 centers following 21,740 patients with IBD (2.39 cases of lymphoma/1000 patients with IBD). The median age at diagnosis of lymphoma was 59 years old (IQR 48–67). The characteristics of the included patients are shown in Table 1. Specific types of lymphoma are presented in Table 2. The median time since IBD diagnosis to diagnosis of lymphoma was 12 years (IQR 5–18 years).

### 3.1. Immunosuppressive and Biological Treatments before Lymphoma Diagnosis

A total of 31 patients (60%) received thiopurines as treatment for their IBD for a median of 47 months (IQR 24–112 months) before diagnosis of lymphoma. Eighteen of these patients had NHL (58%), ten Hodgkin lymphoma (32%), and three T-cell lymphoma (10%).

Twenty patients (38%) were treated with a biological drug before lymphoma diagnosis, but only one of them did not receive thiopurines before (median treatment with thiopurines of 40 months (IQR 23–108)). All of them received an anti-TNF drug as the first biological drug for treating their IBD: 16 (80%) infliximab and 4 adalimumab. Only one patient had been treated at onset of lymphoma with a different biologic, ustekinumab, started 23 months before lymphoma, with this patient having been treated previously with combination therapy with thiopurines and infliximab for 26 months. Anti-TNF drugs were administered during a median of 32 months (IQR 12–63 months) before diagnosis of lymphoma. Eighteen patients (35%) were treated with combination therapy of thiopurines and anti-TNF during a median of 22 months (IQR 6–37 months) before the diagnosis of lymphoma.

In those patients who had been treated with thiopurines, the mean age at diagnosis of lymphoma was 53 ± 17 years old vs. Moreover, 63 ± 12 years old in those patients not treated with these drugs before the diagnosis of lymphoma (*p* = 0.02). The mean age at diagnosis of lymphoma was 47 ± 17 years old in those patients treated with anti-TNF before the diagnosis of lymphoma vs. 63 ± 12 years old in those patients not treated with anti-TNF (*p* = 0.01). Neither the duration of exposure to thiopurines nor anti-TNF was correlated with age at diagnosis of lymphoma.

### 3.2. Diagnosis of Lymphoma

The characteristics of the patients at diagnosis of lymphoma are presented in Table 3. The mean time since the onset of symptoms to lymphoma diagnosis was 65 ± 42 days. The presence of symptoms was the main reason for the suspicion of lymphoma (34 patients; 65%), followed by radiological findings (21 patients; 40%), and alterations in routine blood test (11 patients; 21%). Histology was available in 50 patients (96%). Epstein Barr Virus (EBV) serology was available in 11 patients (21%), with eight being positive (73%).

### 3.3. Treatment of Lymphoma

Most patients were treated with chemotherapy (40 patients: 77%). In those patients with non-Hodgkin lymphoma, the standard treatment with R-CHOP (rituximab, cyclophosphamide, doxorubicin, vincristine, and prednisone) was used in 20 patients (80% of patients receiving chemotherapy), with intrathecal chemoprophylaxis being needed in two patients. In those patients with Hodgkin lymphoma, ABVD (doxorubicin, bleomycin, vinblastine, dacarbazine) was the most common chemotherapy (nine patients: 82%). Three patients (75%) with T-cell lymphoma were treated with chemotherapy. Four patients with lymphoma (7.7%) did not receive any treatment because of poor clinical status. Treatment was unknown/not reported in five patients (10%).

### 3.4. Treatment and Evolution of IBD after Lymphoma Diagnosis

After the diagnosis of lymphoma, IBD treatment was changed in 30 patients (58%). At time of lymphoma diagnosis, 21 patients received thiopurines (40%), withdrawing the drug in all of them after lymphoma diagnosis; 13 patients (25%) were in treatment with biologics at lymphoma diagnosis, withdrawing the drug in 12 of them (92%), maintaining ustekinumab treatment in one patient after lymphoma diagnosis. In 40 cases (77%), there was a consensus between IBD Units and Hematology Units regarding the IBD treatment after lymphoma.

Median follow-up after lymphoma diagnosis was 57 months (IQR 39–102 months). Most patients (30 patients, 58%) did not receive any treatment for IBD after lymphoma diagnosis. Eight patients (15%) were treated with biological therapies (two infliximab, two adalimumab, four ustekinumab and four vedolizumab) after a median of 2.5 months (IQR 0–28 months) since lymphoma diagnosis. Immunomodulators were used in seven patients (13%) for IBD treatment after lymphoma diagnosis in all of them with consensus with Hematology Units: three patients received thiopurines (5.8%) and four (7.7%) methotrexate.

After lymphoma diagnosis, 17 patients (33%) presented an IBD flare, with seven of them (13%) requiring hospital admission. Those patients in whom immunosuppressants or biologicals were reintroduced after lymphoma diagnosis experienced IBD flare more frequently than those patients without these treatments (75% vs. 20%; *p* = 0.01). Eight patients (15%) needed surgery for their IBD after lymphoma: four patients with CD (17% of patients with CD) and four patients with UC (15% of patients with UC).

### 3.5. Relapse of Lymphoma

There was a relapse of lymphoma in five patients (9.6% of patients with lymphoma). The incidence of relapse was 1.7 cases/100 patient-years (95% CI 0.7–4.0 cases/100 patient-years), with a median of 38 months (IQR 23–84 months) since initial lymphoma diagnosis to relapse diagnosis. Chemotherapy was needed in four patients (80%) with relapse of lymphoma, bone marrow transplantation in three (60%), and radiotherapy was required in one patient (20%). Relapse was not related to the type of IBD, sex, smoking habit, specific type of lymphoma (NHL vs. Hodgkin lymphoma), nor with the use and duration of thiopurines or biologics therapies.

### 3.6. Mortality

**Figure 1:**
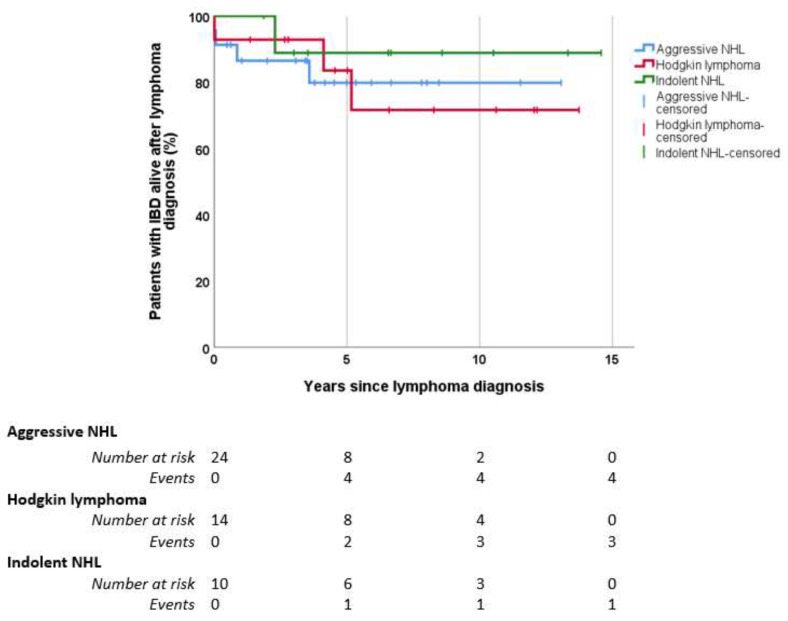
Survival Kaplan–Meier curve of patients with IBD after lymphoma diagnosis. IBD: inflammatory bowel disease. NHL: non-Hodgkin lymphoma. Aggressive NHL: diffuse large B-cell lymphoma, mantle cell lymphoma, plasmablastic lymphoma, and cutaneous lymphoma. Indolent NHL: follicular, marginal zone, and lymphoplasmacytic lymphoma. Five-year survival rate: 80% aggressive NHL, 84% Hodgkin lymphoma, 89% indolent NHL. Ten-year survival rate: 80% aggressive NHL, 72% Hodgkin lymphoma, 89% indolent NHL. Log-rank *p* > 0.05.

Nine patients (17%) died, five with an aggressive NHL (diffuse large B-cell lymphoma), three with Hodgkin lymphoma, and one with an indolent NHL (follicular lymphoma), after a median of 19 months since the diagnosis of lymphoma (IQR 0–48 months). There were no differences in the cumulative survival curve between patients with aggressive NHL lymphoma, indolent NHL, and Hodgkin lymphoma (Figure 1). The cause for death was the lymphoma in four patients (44%), an infection in another four patients (one of them by COVID-19), and one patient died because of postsurgical complications after IBD surgery. Of those patients who died during the follow-up, seven were men (78%), six had UC (22% of patients with UC), and three had CD (13% of them). The mean age at death was 72 ± 12 years old. Four of the patients who died (44%) were on treatment with thiopurines before lymphoma diagnosis for a median of 88 months (IQR 23–158 months), and one of them received biologics monotherapy. Type of IBD, sex, smoking habit, specific type of lymphoma (NHL vs. Hodgkin lymphoma), relapse of lymphoma, having received thiopurines or biologics, and a longer exposition to these therapies were not related with mortality.

## 4. Discussion

To the best of our knowledge, our series is the largest one analyzing the clinical presentation, management, and evolution of lymphoma in patients with IBD, including 52 cases with lymphoma in 21,740 patients with IBD (2.39 cases of lymphoma/1000 patients with IBD). Although an increased risk of lymphoma has been described in patients with CD, in our series, most of the patients had UC, showing the importance of considering this disease in all the patients with IBD [10]. In a recently published series including 653 patients with at least 12 months of cumulative thiopurine monotherapy exposure, five lymphomas were diagnosed, with a median age of 56 years and a median thiopurine duration of 5.4 years [11]. In our series, we found a median age at diagnosis of lymphoma of 59 years, and a median of 47 months with thiopurines before the diagnosis of lymphoma. We found that around 60% of patients had been treated with immunosuppressive drugs and/or anti-TNF before the diagnosis of lymphoma. Therefore, it is also important to advise that in clinical practice, an important proportion of lymphomas could be diagnosed in those patients without previous treatment with immunosuppressive or anti-TNF drugs. Although in Western countries, several studies have reported an approximately four-fold higher risk of lymphoma in IBD patients treated with thiopurines, some series in Asia have not found an increased risk of lymphoma in IBD patients treated with thiopurines, with or without an anti-TNF agent, maybe because of a lower baseline incidence of lymphomas, a lower standard dose of thiopurines used in countries like Japan compared with Western countries, and/or genetic factors [12,13]. In a recent systematic review and meta-analysis including four observational cohort studies totaling 261,689 IBD patients an incidence rate of lymphomas of 0.34 per 1000 patient-years (95% CI: 0.30–0.37) was found in patients unexposed to either anti-TNF or thiopurines [14]. In this study, an increased risk of lymphoma was found in those patients exposed to these drugs, with a pooled incidence rate ratio (IRR) of 1.52 (95% CI: 1.06–2.19) for those patients exposed to anti-TNF monotherapy vs. patients unexposed to anti-TNF of thiopurines; IRR 2.23 (95% CI: 1.79–2.79) in patients exposed to thiopurines monotherapy vs. controls; and IRR 3.71 (95% CI: 2.30–6.00) for patients on combination therapy vs. controls.

Vedolizumab and ustekinumab do not appear to correspond to an increased risk of lymphoma [15]. In our series, only one patient was on treatment with ustekinumab at the onset of lymphoma but had been treated previously with combination therapy of thiopurines and anti-TNF. Because of the low incidence of individual cancers, the assessment of an increased cancer risk related to new IBD drugs requires a total observation time of 20,000–50,000 patient-years, with pharmacoepidemiology being very important [16].

Lymphoma commonly presents as painless adenopathy [17]. Radiological examinations and lymph node or mass biopsy are usually performed for the diagnosis of lymphoma. Currently, a bone marrow biopsy is only recommended for diffuse large B-cell lymphoma with a negative PET-CT result [7]. General practice is to treat patients on the basis of either limited (stages I and II, nonbulky) or advanced (stages III or IV) disease, usually with chemotherapy alone or in combination with radiotherapy [7,18,19]. A Japanese study analyzed 15 cases with IBD-associated lymphoid and myeloid proliferative disorder [20]. They recommend that low-grade and high-grade lymphomas should be clearly distinguished, because these conditions showed different clinicopathological features in terms of histology, EBV infection status, and prognosis, with low-grade lymphomas usually being related to an indolent clinical course and favorable prognosis, whereas high-grade lymphoproliferative disorders require chemotherapy and multidisciplinary treatments, with a shorter survival time.

The WHO classification system identifies multiple different subtypes of lymphoma [6]. In our series, as had been previously described in other IBD series and in the general population, diffuse large B-cell lymphoma was the most frequent type of lymphoma [21]. In any case, a wide histological variety of lymphomas has been described in patients with IBD, mainly EBV-associated lymphomas, including some rare but aggressive lymphomas such as hepatoesplenic T cell lymphomas or plasmablastic lymphoma [22,23]. Hodgkin lymphoma peaked in people aged 75 to 79 (2016–2018), with a 5-year survival rate of 82% (2013–2017) in the UK [24]. The classification of NHL is complex, with the peak incidence rate having been described as being in people aged 80 to 84 in the UK between 2016 and 2018, and a 5-year survival rate of 66% between 2013 and 2017 [24]. In the United States, the SEER program reported a 5-year relative survival of 89% for Hodgkin lymphoma, 74% for NHL, and 65% for specific diffuse large B-cell lymphoma between 2012 and 2018 [25]. Our series shows a similar 5-year survival rate to these population programs in patients with Hodgkin lymphoma (84%). This is in line with previous data that do not show a worse prognosis of lymphomas in IBD patients [1]. Genetic determinants, geographic and temporal variability, obesity, and some infections such as EBV or hepatitis C virus can increase or modulate the risk of NHL in the general population [26,27]. In the majority of countries, the incidence rates of NHL increased until the 1990s, followed by a stabilization at that point, when taking into account data circa 1980–2012 [28]. Data from the prospective epidemiological registry of lymphoid neoplasms in Spain (RELINF) shows a distribution of the most frequent lymphomas similar in our country to those reported in Europe and in the USA [29].

In our series, all patients treated with thiopurines and/or anti-TNF were withdrawn from these drugs after lymphoma diagnosis, although in some patients, these drugs were administered after diagnosis of lymphoma, mainly in those patients with IBD flares, in the presence of a case-by-case consensus with Hematology Units. It is usually recommended to stop and not initiate or resume drug classes with established promoting effects on the diagnosed type of cancer. However, in some cases, because of IBD evolution and the lack of other treatment alternatives, these drugs may be used in agreement with the oncologist and the patient, although with the increasing number of options available for IBD treatment, this will become increasingly rare [16,30,31].

After remission of the lymphoma, surveillance is necessary in order to detect relapse. Lifetime relapse of lymphoma in the general population occurs in 10 to 40% of patients [17]. In our series, the relapse rate was 9.6%. Patients older than 60 years at diagnosis of lymphoma have worse outcomes [17]. In our series, nine patients (17%) died, with a mean age of 72 ± 12 years. Infections were a common cause of death in these patients.

Our study has several limitations. The true incidence of lymphomas may be underestimated because it is not a required field in the ENEIDA registry; however, because of the importance of this pathology in the management of IBD, the number of non-reported lymphomas is probably very low. Infection with EBV is a risk factor for the development of lymphoma, mainly Hodgkin and Burkitt lymphomas, with a risk of developing an aggressive lymphoproliferative disease in case of being infected with EBV at the time of thiopurine therapy, although it is unclear if and how EBV contributes to lymphoma risk in IBD patients on immunosuppression [15,32,33]. Long-term monitoring of EBV viral load, as determined by polymerase chain reaction (PCR), should be performed in immunosuppressed patients, as this is a suspected risk factor for lymphoma development [34]. Currently, EBV infection status is available in almost all IBD patients, but previously, EBV infection status was not always routine in clinical practice. In our series, most patients did not have EBV status before diagnosis of lymphoma, although considering EBV seroprevalence in adults with IBD, it can be suspected that it would be >80% [33].

## 5. Conclusions

Lymphoma in patients with IBD is rare. It is important to consider this disease in all IBD patients, even in those patients without treatment with thiopurines or anti-TNF. At diagnosis of lymphoma, it is recommended to consider the need for changes in IBD treatment, and a multidisciplinary approach to lymphoma and IBD together with the Hematology Unit. After treatment for lymphoma, a long-term follow-up is needed due to the possibility of lymphoma relapse. Lymphoma itself and infections are the most frequent causes of mortality in these patients.

## Figures and Tables

**Table 1:** Characteristics of the patients. IBD: inflammatory bowel disease. IQR: interquartile range. * Forty-three patients had this data available.

	Number of Patients (%)
**Men**	35 (67)
**Smokers or former smokers**	22 (51) *
**Type of IBD**	
Ulcerative colitis	27 (57)
Extensive colitis	12 (44)
Left-sided colitis	9 (33)
Proctitis	6 (22)
Crohn’s disease	23 (44)
L1. Ileal	5 (22)
L2. Colonic	13 (56)
L3. Ileocolonic	5 (22)
p. Perianal disease	8 (35)
Unclassified colitis	2 (3.9)
**Median age at diagnosis of IBD**	45 years (IQR 28–57)
**Median age at diagnosis of lymphoma**	59 years (IQR 48–67)

**Table 2:** Specific types of lymphoma.

**Non-Hodgkin lymphoma:** 34 patients (**65**%) -Diffuse large B-cell lymphoma: 19 (36%) -Follicular lymphoma: 8 (15%) -Other: 7 (2 mantle cell lymphoma, 2 plasmablastic lymphoma, 1 cutaneous lymphoma, 1 lymphoplasmacytic lymphoma, 1 marginal zone lymphoma) **Hodgkin lymphoma:** 14 patients (27%) **T-cell lymphoma:** 4 patients (7.7%)

**Table 3:** Characteristics of the patients at diagnosis of lymphoma. NHL: Non-Hodgkin lymphoma.

	Number of Patients (%)
**Symptoms at diagnosis of lymphoma**	
Adenopathy or mass	20 (38)
Fever	13 (25)
Weight loss	13 (25)
Asthenia	12 (23)
Abdominal pain	6 (11)
Anorexia	5 (10)
Profuse sweating	5 (10)
Pruritus	2 (4)
Asymptomatic	8 (15)
**Location of adenopathy or mass**	
Cervical	13 (65)
Inguinal	4 (20)
Supraclavicular	3 (15)
Mediastinal	3 (15)
Retroperitoneal	2 (10)
Axillar	2 (10)
**Ann Arbor stage**	
*Aggressive NHL* (*n* = 24)	
I	6 (25)
II	1 (4)
III	0 (0)
IV	15 (63)
Unknown	2 (8)
*Hodgkin lymphoma* (*n* = 14)	
I	1 (7)
II	3 (21)
III	4 (29)
IV	3 (21)
Unknown	3 (21)
*Indolent NHL* (*n* = 10)	
I	2 (20)
II	2 (20)
III	0 (0)
IV	4 (40)
Unknown	2 (20)

## Data Availability

The data presented in this study are available on request from the corresponding author. The data are not publicly available due to privacy reasons.
